# ZPD-2, a Small Compound That Inhibits α-Synuclein Amyloid Aggregation and Its Seeded Polymerization

**DOI:** 10.3389/fnmol.2019.00306

**Published:** 2019-12-17

**Authors:** Samuel Peña-Díaz, Jordi Pujols, María Conde-Giménez, Anita Čarija, Esther Dalfo, Jesús García, Susanna Navarro, Francisca Pinheiro, Jaime Santos, Xavier Salvatella, Javier Sancho, Salvador Ventura

**Affiliations:** ^1^Institut de Biotecnologia i Biomedicina, Universitat Autonoma de Barcelona, Barcelona, Spain; ^2^Departament de Bioquimica i Biologia Molecular, Universitat Autonoma de Barcelona, Barcelona, Spain; ^3^Department of Biochemistry and Molecular and Cell Biology, Institute for Biocomputation and Physics of Complex Systems (BIFI), University of Zaragoza, Zaragoza, Spain; ^4^Faculty of Medicine, M2, Universitat Autonoma de Barcelona, Barcelona, Spain; ^5^Faculty of Medicine, University of Vic – Central University of Catalonia, Vic, Spain; ^6^Institute for Research in Biomedicine, The Barcelona Institute of Science and Technology, Barcelona, Spain; ^7^Catalan Institute for Research and Advance Studies, Barcelona, Spain

**Keywords:** Parkinson’s disease, α-synuclein, amyloid, protein aggregation, aggregation inhibitor, *Caenorhabditis elegans*, neurodegeneration

## Abstract

α-Synuclein (α-Syn) forms toxic intracellular protein inclusions and transmissible amyloid structures in Parkinson’s disease (PD). Preventing α-Syn self-assembly has become one of the most promising approaches in the search for disease-modifying treatments for this neurodegenerative disorder. Here, we describe the capacity of a small molecule (ZPD-2), identified after a high-throughput screening, to inhibit α-Syn aggregation. ZPD-2 inhibits the aggregation of *wild-type* α-Syn and the A30P and H50Q familial variants *in vitro* at substoichiometric compound:protein ratios. In addition, the molecule prevents the spreading of α-Syn seeds in protein misfolding cyclic amplification assays. ZPD-2 is active against different α-Syn strains and blocks their seeded polymerization. Treating with ZPD-2 two different PD *Caenorhabditis elegans* models that express α-Syn either in muscle or in dopaminergic (DA) neurons substantially reduces the number of α-Syn inclusions and decreases synuclein-induced DA neurons degeneration. Overall, ZPD-2 is a hit compound worth to be explored in order to develop lead molecules for therapeutic intervention in PD.

## Introduction

Parkinson’s disease (PD) is a neurodegenerative disorder that affects about 0.3% of the population and >1% of people over 60 years of age (4% over 80 years) (Nussbaum and Ellis, 2003; Dexter and Jenner, 2013). It is characterized by the loss of dopaminergic (DA) neurons in *substantia nigra pars compacta*, which compromises the motor capacity of PD-suffering patients, producing tremor, rigidity, and bradykinesia (Marti et al., 2003). Additionally, since the disease spreads to the cerebral cortex (Braak et al., 2003), symptoms could include emotional and cognitive impairment (Marti et al., 2003). Nowadays, treatments are focused on alleviating the above mentioned motor symptoms, mostly using dopamine replacement by administration of dopamine precursor (L-DOPA), combined with carbidopa, a L-DOPA decarboxylase inhibitor, and/or catechol-*O*-methyl transferase inhibitors and monoamine oxidase-B inhibitors (Dexter and Jenner, 2013). However, these treatments do not prevent the progression of PD and they lose efficacy as the disease advances.

Parkinson’s disease is pathologically characterized by the accumulation of protein aggregates in the neuronal body, Lewy’s bodies (LB), and/or fibrils deposited in neuronal processes, Lewy’s neurites (LN), of affected neurons (Spillantini et al., 1997). These inclusions are mainly composed of α-synuclein (α-Syn), a protein predominantly expressed in the synaptic termination of DA neurons (Bendor et al., 2013). This evidence, together with the identification of mutations in the gene that encodes for this protein (SNCA) as the cause behind familial cases of PD (Polymeropoulos et al., 1997) and the observation that duplications and triplications of the SNCA gene lead to highly penetrant forms of the disease (Singleton et al., 2003; Ibanez et al., 2004) directly connect PD and α-Syn. In fact, the presence of aggregated α-Syn in the brain is a common feature of a group of diseases named synucleinopathies, which, in addition to PD, include Dementia with Lewy’s bodies (DLB) and multiple system atrophy (MSA), among others (Marti et al., 2003).

In solution, α-Syn is a 140 amino acid intrinsically disordered protein whose function seems to be related with vesicle trafficking (Bendor et al., 2013). *In vitro* it forms thermodynamically stable amyloid aggregates (Serpell et al., 2000) that can display different conformational features (Li et al., 2018). The formation of amyloids by α-Syn follows the typical sigmoidal kinetics, reflecting a nucleation-polymerization process (Sabate et al., 2003); although secondary nucleation reactions might also occur (Xue et al., 2010). *In vivo*, α-Syn assemblies exert a toxic effect (Winner et al., 2011) and could be transmitted from cell to cell in a prion-like manner by seeding native α-Syn aggregation in previously unaffected neurons (Hansen et al., 2011).

**FIGURE 1:**
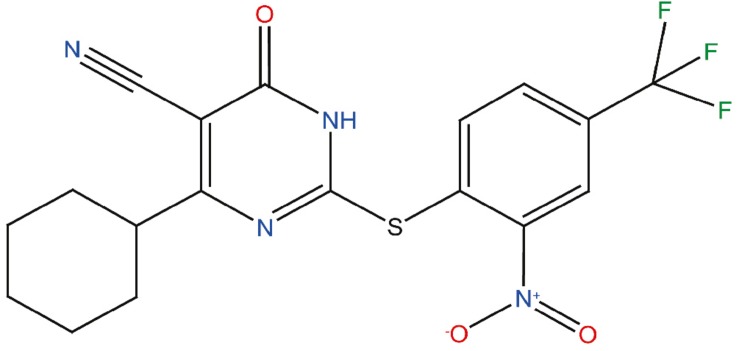
Chemical structure of the compound ZPD-2. ZPD-2 corresponds to 4-cyclohexyl-2-((2-nitro-4-(trifluoromethyl)phenyl)thio)-6-oxo-1,6-dihydropyrimidine-5-carbonitrile.

Preventing α-Syn aggregation seems to hold the potential to achieve significant therapeutic impact. Several strategies have been developed toward this objective: SNCA gene-silencing approaches to decrease the protein levels (McCormack et al., 2010), methods to increase the clearance of aggregated α-Syn by autophagic and proteasomal machineries (Gao et al., 2019), and molecules intended to avoid the formation and/or propagation of aggregated α-Syn (Dehay et al., 2015; Hauser, 2015). One of the main limitations of this last strategy is the absence of a well-defined structure of monomeric α-Syn in solution, due to its intrinsically disordered nature, which hampers the rational design of inhibitors. High-throughput screening protocols have been developed to circumvent this problem (Silva et al., 2011; Levin et al., 2014). A number of promising small molecules have been discovered with this approach, including anle138b (Levin et al., 2014), BIOD303 (Moree et al., 2015), fasudil (Tatenhorst et al., 2016), squalamine (Perni et al., 2017), or SynuClean-D (SCD) (Pujols et al., 2018). In this context, we have developed a robust screening and validation protocol to analyze large chemical libraries in the search for effective inhibitors of α-Syn aggregation (Pujols et al., 2017). The *in vitro* pipeline integrates thioflavin-T (Th-T) fluorescence and light scattering measurements, transmission electron microscopy (TEM), and protein misfolding cyclic amplification assays (PMCA). This approach allowed us to identify ZPD-2 (Figure 1) as a novel small molecule able to inhibit the aggregation of *wild-type* (WT) α-Syn, as well as that of the A30P (Kruger et al., 1998) and H50Q (Appel-Cresswell et al., 2013) familial mutants, being active against the seeded polymerization of different α-Syn strains. The compound displayed low toxicity for neuronal human cells and demonstrated significant inhibitory capacity in two well-established *Caenorhabditis elegans* models of PD (van Ham et al., 2008; Harrington et al., 2012).

## Materials and Methods

### Protein Purification

Protein expression and purification of WT α-Syn and its variants (H50Q and A30P) were carried out as previously described (Pujols et al., 2017) and the resulting purified protein was lyophilized and kept at −80°C until its use.

### *In vitro* Aggregation of α-Syn

α-Syn was resuspended in sterile PBS and filtered through 0.22 μm membranes to remove small aggregates. Aggregation was performed in a sealed 96-well plate, containing 70 μM α-Syn (WT, A30P or H50Q), 40 μM Th-T in PBS 1×, a 1/8″ diameter Teflon polyball (Polysciences Europe GmbH, Eppelheim, Germany) and 100 μM ZPD-2 or DMSO (in control samples) in a total volume of 150 μL per well. The plate was incubated at 100 rpm and 37°C after having been fixed in an orbital culture shaker Max-Q 4000 (ThermoScientific, Waltham, MA, United States). Measurements of Th-T fluorescence were done every 2 h in a Victor3.0 Multilabel Reader (PerkinElmer, Waltham, MA, United States), exciting through a 430–450 nm filter and collecting the emission signal with a 480–510 filter. Each assay was done in triplicate. The values of the aggregation kinetics were fitted to the following Eq. 1 (Crespo et al., 2016):

(1) $$\propto =1-\frac{1}{k_{\text{b}}\,\left(e^{k_{\text{a}}\,t}-1\right)+1}$$

where *k*_b_ and *k*_a_ constitute the homogeneous nucleation rate constant and the secondary rate constant (fibril elongation and secondary nucleation), respectively (Crespo et al., 2016).

Titration assays were done by applying different ZPD-2 concentrations (200, 150, 100, 75, 50, 25, and 10 μM). Time-dependent assays were developed by adding 100 μM of ZPD-2 at different time points after the beginning of the reaction (4, 8, 12, 16, 20, and 24 h). In all cases a fixed concentration of α-Syn at 70 μM was maintained.

Strains were generated as previously described (Bousset et al., 2013; Peelaerts et al., 2015; Carija et al., 2019). Briefly, lyophilized α-Syn was resuspended in PBS 1× and dialyzed for 24 h in a 1:1000 (v/v) ratio with buffer B (50 mM Tris–HCl pH 7.0), or buffer C (50 mM Tris–HCl pH 7.0 supplemented with 150 mM NaCl). Then, the protein was filtered through 0.22 μm membrane and incubated at 70 μM in presence or absence of 100 μM ZPD-2 in a 96-well plate as described above. For the seeding assays, α-Syn pre-formed fibrils were sonicated for 5 min and then added to the aggregation reaction at ratios of 1% (v/v) for each condition. The plate was then incubated, and Th-T fluorescence measured as previously indicated.

The soluble fraction was obtained for subsequent quantification by centrifuging 300 μL of aggregated sample at 16,900 × *g* for 90 min. The supernatant was then recovered and loaded into a Tricine–SDS-PAGE gel. Gels were stained with Blue safe. Finally, the density of the α-Syn bands was calculated using Quantity One software (Bio-Rad, Hercules, CA, United States). Experiments were done at least in triplicate.

### Transmission Electron Microscopy

End-point α-Syn aggregates incubated for 32 h were collected, diluted 1:10 with PBS 1× and sonicated for 5 min. Five microliters of these sonicated samples was placed rapidly on a carbon-coated copper grid and incubated for 5 min. The grids were dried with a filter paper to withdraw the excess of sample and immediately washed twice with miliQ water. Finally, 5 μL of 2% (w/v) uranyl acetate was added to the top of the grid and incubated for 2 min. The excess of uranyl acetate was removed with a filter paper and grids were left to air-dry for 10 min. Images were obtained using a TEM Jeol 1400 (Peabody, MA, United States) operating at an accelerating voltage of 120 kV. A minimum of 30 fields were screened per sample, in order to collect representative images.

### Light Scattering

End-point α-Syn aggregates were collected, placed into a quartz cuvette, and analyzed in a Cary Eclipse Fluorescence Spectrophotometer (Agilent, Santa Clara, CA, United States). The sample was excited at 300 nm and the subsequent scattering at 90° monitored between 280 and 320 nm.

### Protein Misfolding Cyclic Amplification

The PMCA assay was carried out as previously described (Herva et al., 2014). Briefly, α-Syn was resuspended to a final concentration of 90 μM in Conversion Buffer (PBS 1×, 1% Triton X-100, 150 mM NaCl), supplemented with Complete Protease Inhibitor Mixture (Roche Applied Science, Penzberg, Germany). Sixty microliters of this α-Syn solution was added into 200-μL PCR tubes containing 1.0 mm silica beads (Biospec Products, Bartlesville, OK, United States). Samples were exposed to 24-h cycles of 30 s sonication and 30 min incubation at 37°C, using a Misonix 4000 sonicator, at 70% power. After every 24 h-cycle, 1 μL of the incubated sample was added to a new PCR-tube containing fresh α-Syn. This process was repeated for 5 days. In the case of treated samples, ZPD-2 was added in each cycle to the fresh non-sonicated sample to a final concentration of 128 μM, which corresponds to the 0.7:1 α-Syn:ZPD-2 ratio of the previous set of aggregation kinetics assays. Untreated samples were prepared adding the same concentration of DMSO (0.26%) present in the treated mixtures. All the reactions were made in triplicate.

At the end of each cycle, 10 μL of the incubated samples were diluted 1:10 with 90 μL of PBS 1×, 40 μM Th-T. Th-T fluorescence was measured in a Cary Eclipse Fluorescence Spectrophotometer (Agilent, Santa Clara, CA, United States), exciting at 445 nm and collecting the emission signal between 460 and 600 nm.

### Proteinase K Digestion

For protein digestion, 6 μL of Proteinase K (5 μg/mL final concentration) was added to 18 μL of PMCA aggregated samples and incubated for 30 min at 37°C. After the incubation, 8 μL of loading buffer containing 1% β-mercaptoethanol was added and the enzyme was thermally inactivated at 95°C for 10 min. Finally, 7 μL of the incubated and stained samples was loaded into a Tricine–SDS-PAGE gel together with unstained Protein Standard markers (ThermoFisher Scientific, Waltham, MA, United States). Gels were stained with Blue safe.

### Nuclear Magnetic Resonance

Expression of ^15^N-labeled human WT α-Syn was carried out in *Escherchia coli BL21 DE3* strain. First, cells were grown in LB medium until an OD_600_ of 0.6. The culture was then centrifuged at 3000 rpm for 15 min and the pellets collected and resuspended in 1 L minimal medium, composed of: 768 mL of miliQ water with 1 mL of ampicillin 100 mg/mL, 100 μL CaCl_2_ 1 M, 2 mL MgSO_4_ 2 M, 20 mL glucose 20%, 10 mL vitamins 100× (Sigma–Aldrich, Darmstadt, Germany), 200 mL salts M9, and 1 g ^15^NH_4_ (Cambridge Isotope Laboratories, Inc., Tewksbury, MA, United States). Cells were incubated for 1 h at 37°C and 250 rpm. After that, protein expression was induced for 4 h with 1 mM IPTG. Protein was purified as previously described (Pujols et al., 2017).

^1^H-^15^N HSQC spectra were obtained at 20°C on a Bruker 600 MHz NMR spectrometer equipped with a cryoprobe in a mixture containing 70 μM ^15^N-labeled α-Syn, PBS buffer (pH 7.4), 2.5% d6-DMSO, and 10% D_2_O in the absence or in the presence of 100 μM ZPD-2.

### Toxicity Assays

Neuroblastoma cells were incubated 24 h in DMEM medium in a 96-well plate before the addition of different concentrations of ZPD-2 (from 1 μM to 1 mM). Cells were incubated for 48 h at 37°C and PrestoBlue^®^ reagent (ThermoFisher Scientific, Waltham, MA, United States) was added to analyze cell death. Treated and untreated cells were incubated with PrestoBlue^®^ for 10 min at 37°C. Finally, fluorescence emission was measured by exciting at 560 nm and collecting at 590 nm.

### *Caenorhabditis elegans* Assays

#### Maintenance

Animals synchronization was carried out by bleaching and overnight hatching in M9 (3 g/L KH_2_PO_4_, 6 g/L Na_2_HPO_4_, 5 g/L NaCl, 1 M MgSO_4_) buffer. Thus, nematodes were cultured at 20°C on growth media plates (NGM) containing 1 mM CaCl_2_, 1 mM MgSO_4_, 5 μg/mL cholesterol, 250 M KH_2_PO_4_ pH 6.0, 17 g/L Agar, and 3 g/L NaCl. Plates were previously seeded with *E. coli OP50* strain. Nematodes were maintained using standard protocols (Brenner, 1974).

#### Strains

Strain NL5901, *unc-119(ed3)* III; *pkIs2386 [Punc-54:*α*-SYN:YFP; unc-119(*+)] was obtained from the *C. elegans* Genetic Center (CGC). For the α-Syn-induced DA degeneration analysis, strain UA196 (Harrington et al., 2012), gifted generously by the laboratory of Dr. Guy Caldwell (Department of Biological Science, The University of Alabama, Tuscaloosa, AL, United States), was used; [*sid-1(pk3321)*; *baIn33* (*Pdat-1:sid-1*, *Pmyo-2:mCherry*); *baIn11* (*Pdat-1:*α*-SYN; Pdat-1:GFP*)]. In the main text, this strain was named *Pdat-1:GFP; Pdat-1*:α*-SYN*.

#### ZPD-2 Administration

After cooled, the autoclaved NGM agar medium (1 mM CaCl_2_, 1 mM MgSO_4_, 5 μg/mL cholesterol, 250 M KH_2_PO_4_ pH 6.0, 17 g/L Agar, and 3 g/L NaCl) was enriched with 100 μM of a stock of ZPD-2 in 0.2% DMSO to a final concentration of 10 μM. After 2 days, plates were seeded with 250 μL of *E. coli OP50* with 10 μM of ZPD-2. Nematodes were placed on the plates at larval stages L4 and exposed either to ZPD-2 or DMSO (controls) for 7 days. Daily transfer was done to avoid cross progeny.

#### Aggregate Quantification

The number of cellular inclusions was quantified as previously described (van Ham et al., 2008; Munoz-Lobato et al., 2014). Briefly, NL5901 (*Punc-54:*α*-SYN:YFP*) worms were age-synchronized and left overnight to hatch. Nematodes in phase L1 were cultured and grown into individual NGM plates seeded with *E. coli OP50*. When animals reached L4 developmental stage, they were transferred onto either ZPD-2-treated plates or DMSO-treated plates (negative control). Every day, animals were transferred into a new plate to avoid cross contamination. At stage L4 + 7, the aggregates in the anterior part of every single animal were counted. For each experiment, 30 7-day-old nematodes per treatment were analyzed using a Nikon Eclipse E800 epifluorescence microscope equipped with an Endow GFP HYQ filter cube (Chroma Technology Corp., Bellows Falls, VT, United States) and each experiment was carried out in triplicate. Inclusions could be described as discrete bright structures, with edges distinguishable from surrounding fluorescence. ImageJ software was used for measuring the number of cellular aggregates considering the area dimensions. For the quantification of α-syn aggregates in *C. elegans* one single image was taken from each animal. Every image contained among 30–45 stacks (1 μm) that allowed to detect aggregates at different animal positions. At least 30 animals were imaged for each assayed condition.

#### *C. elegans* Lifespan Analysis

L4-stage synchronized *C. elegans* were exposed to 10 μM of ZPD-2 or DMSO (controls) during lifespan analysis. The worms were classified as alive, dead, or censored every 2 days by determining their movement and response to nose and tail tap. The numbers of alive and dead worms were recorded until all worms perished. The data were plotted as a Kaplan–Meier survival curve and groups compared using a Wilcoxon-test.

#### *C. elegans* Neurodegeneration Assays

Worms were analyzed for α-Syn-induced DA neurodegeneration as described previously (Harrington et al., 2012). Briefly, 20–30 L4-staged animals were transferred to ZPD-2 – NGM plates and make them grow up to 7 days (L4 + 7 days of development) after which the DA cell death induced by the over-expression of α-Syn was analyzed by fluorescence. Plates containing only 0.2% DMSO, without ZPD-2, were used as control. Worms were transferred daily to avoid cross contamination.

The six anterior DA neurons (four CEP and two ADE DA neurons) were scored for neurodegeneration according to previously described criteria (Sulston et al., 1975; Harrington et al., 2012). Worms were considered normal when all six anterior DA neurons (four CEP, cephalic, and two ADE, anterior deirid) were present without any visible signs of degeneration. If a worm displayed degeneration in at least one of the six neurons, it was scored as exhibiting degeneration. For each independent experiment, 30 worms of each treatment were examined under a Nikon Eclipse E800 epifluorescence microscope equipped with an Endow GFP HYQ filter cube (Chroma Technology Corp., Bellows Falls, VT, United States).

#### Microscopy and Imaging

Animals were placed in a 1 mM solution of sodium azide and mounted with a coverslip on a 4% agarose pad. Animals were visualized with a Nikon Eclipse E800 epifluorescence microscope. The system acquires a series of frames at specific *Z*-axis position (focal plane) using a *Z*-axis motor device. Animals were examined at 100× magnification to examine α-Syn-induced DA cell death and at 40× to examine α-Syn apparent aggregate.

### Statistical Analysis

All graphs were generated with GraphPad Prism 6.0 software (GraphPad Software Inc., La Jolla, CA, United States). Data were analyzed by two-way ANOVA Tukey’s HSD test using SPSS software version 20.0 (IBM Analytics, Armonk, NY, United States) and *t*-test using GraphPad software version 6.0 (GraphPad Software Inc., La Jolla, CA, United States). All data are shown as means and standard error of mean (SEM). *p* < 0.05 was considered statistically significant. In the graphs ^∗^, ^∗∗^, and ^∗∗∗^ indicate *p* < 0.05, *p* < 0.01, and *p* < 0.001, respectively.

## Results

### ZPD-2 Reduces and Delays the Aggregation of Human α-Synuclein *in vitro*

We designed and optimized a screening protocol that allows to follow the aggregation kinetics of α-Syn by monitoring Th-T fluorescence emission for 32 h. This approach permitted us to study the inhibitory potential of more than 14,000 compounds (Pujols et al., 2017, 2018). The activity of molecules able to reduce significantly the final amount of Th-positive material and/or impact the nucleation or elongation rates of the reaction was further confirmed using light scattering and TEM measurements at the end of the reaction. This allowed us to identify 30 active compounds, most of which seem not to be connected in terms of structure, precluding QSAR studies. We have previously described the properties of SCD a molecule that acts preferentially on top of α-Syn proto-fibrillar or fibrillar assemblies (Pujols et al., 2018). Here, we describe the properties of ZPD-2 (Figure 1), a compound that differs in its mechanism of action. SCD and ZPD-2 share a benzotrifluoride group, which suggested that it could constitute the minimal inhibitory unit; however, this group is devoid of any anti-aggregation activity by itself (unpublished), indicating that, most likely, it only acts as a framework for the different active groups in the two molecules.

**FIGURE 2:**
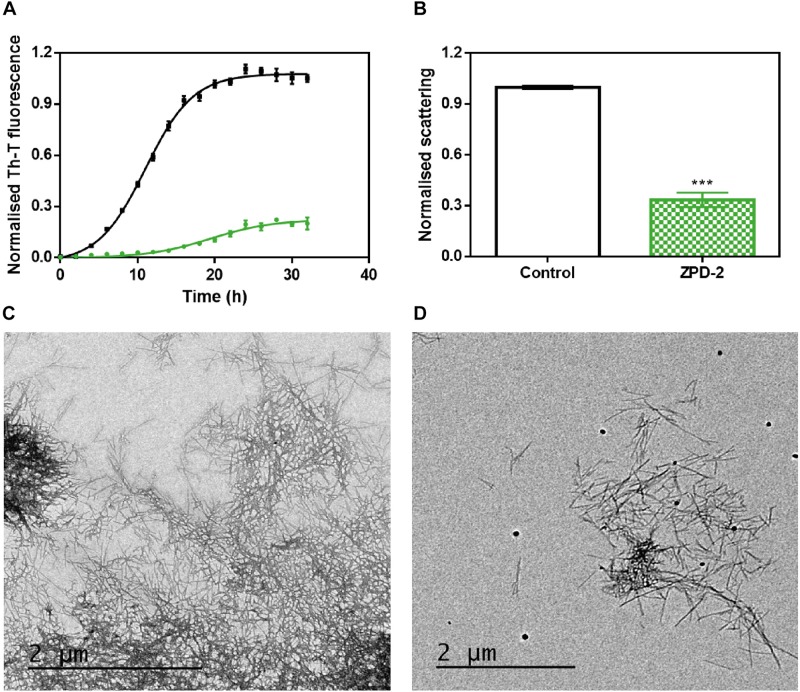
ZPD-2 inhibits the aggregation of wild-type α-synuclein *in vitro*. **(A)** Aggregation kinetics of α-Syn in absence (black) and presence (green) of ZPD-2. Intensity of Th-T fluorescence is plotted as a function of time. **(B)** Light scattering of end-point aggregates is measured at 300 nm for untreated (white) and ZPD-2-treated samples (green). **(C,D)** Representative TEM images of untreated **(C)** and ZPD-2-treated **(D)** samples. Th-T fluorescence is expressed as normalized means. Final points were obtained at 48 h after the aggregation reaction begin. Error bars are shown as standard errors of mean values, ^∗∗∗^*p* < 0.001.

The incubation of 70 μM of α-Syn in the presence and absence of 100 μM of ZPD-2 revealed that the compound modulated the protein aggregation, reducing the formation of Th-T positive structures at the end of the reaction by an 80%, while extending *t*_50_ by 8 h (Figure 2A). The analysis of the kinetics revealed a reduction in the nucleation rate constant in presence of ZPD-2 (*k*_*b*_ = 0.008833) by threefold, when compared to the control reaction (*k*_b_ = 0.02754). The autocatalytic rate constant was also lower in the treated sample (*k*_a_ = 0.2432 h^–1^) than in the control (*k*_a_ = 0.3230 h^–1^). Light scattering measurements at 300 nm confirmed that the observed reduction in Th-T fluorescence corresponds to an effective decrease in the levels of α-Syn aggregates, with a 67% decrease in the dispersion of light in the presence of ZPD-2 (Figure 2B). TEM images corroborated that the samples incubated with ZPD-2 (Figure 2D) contained less fibrils per field than the non-treated ones (Figure 2C). In good agreement with these data, quantification of soluble α-Syn at the end of the aggregation reaction indicated that its level was threefold higher in ZPD-2-treated samples (Supplementary Figure S1A).

**FIGURE 3:**
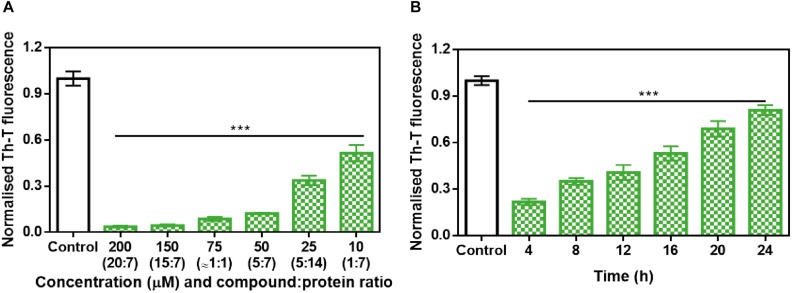
Analysis of the inhibitory capacity of ZPD-2. **(A)** Titration of the effect of different concentrations of ZPD-2 on 70 μM α-Syn aggregation. **(B)** Th-T fluorescence of α-Syn end-point aggregates after the addition of ZPD-2 at different time points during the aggregation kinetics. Th-T fluorescence is plotted as normalized means. End-points were obtained at 48 h of α-Syn incubation. Error bars are shown as standard errors of mean values, ^∗∗∗^*p* < 0.001.

Further analysis of the inhibition capacity of ZPD-2 indicated that it exhibited a dose-dependent effect, displaying a statistically significant effect even at 10 μM (1:7 compound:protein ratio) (Figure 3A), where the final Th-T signal was reduced by 49%.

To address the time window in which ZPD-2 is active, we set up aggregation reactions with a constant amount of ZPD-2 added at different time points after the reaction begins. A time-dependent response was observed (Figure 3B), with a very significant inhibition when ZPD-2 was added at early (4–8 h) and intermediate (12–16 h) times, and a less pronounced effect when it was added at the plateau phase (20–24 h). This indicates that ZPD-2 is mostly active against the species formed early in the aggregation reaction, consistent with its highest impact on the nucleation rate constant *k*_b_. Importantly, NMR studies using isotopically labeled monomeric and soluble α-Syn indicated that ZPD-2 does not interact with its native form, since we could not detect any perturbations in chemical shifts or peak intensities in α-Syn in the presence of a molar excess of the molecule (Supplementary Figure S2).

**FIGURE 4:**
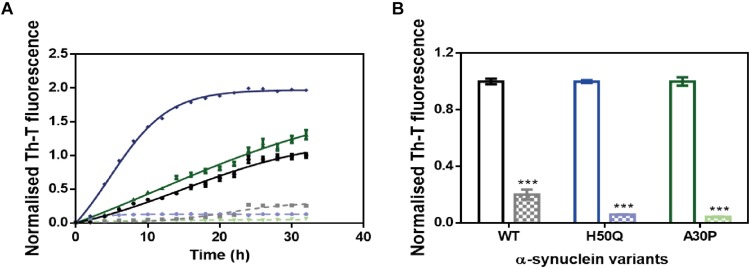
ZPD-2 inhibits the aggregation of α-synuclein familial variants. **(A)** Aggregation kinetics of WT (black), H50Q (blue), and A30P (green) variants of α-Syn in presence (dotted) and absence (continuous) of ZPD-2, using Th-T as reporter. **(B)** End-point measurements of the aggregation of WT, H50Q, and A30P variants of α-Syn in presence (dotted) or absence (continuous) of ZPD-2 Th-T fluorescence are expressed as normalized means. Error bars are shown as standard errors of mean values.

Several α-Syn single point mutations are connected with the onset of familial cases of PD (Kruger et al., 1998; Appel-Cresswell et al., 2013). We studied the ability of ZPD-2 to prevent the aggregation of two of the most frequent and aggressive variants, H50Q and A30P. The molecule was also active against these α-Syn forms in kinetic assays (Figure 4A). According to the relative Th-T signal at the end of the reaction in ZPD-2-treated and non-treated samples, the molecule inhibited the aggregation of A30P and H50Q by 96 and 94%, respectively (Figure 4B).

### ZPD-2 Prevents α-Syn Seeded Aggregation in Protein Misfolding Cyclic Amplification Assays

**FIGURE 5:**
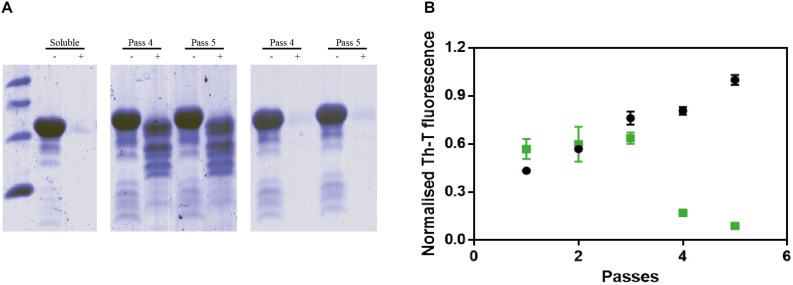
PMCA of α-synuclein in presence of ZPD-2. **(A)** Tricine–SDS-PAGE gels of untreated **(middle)** and ZPD-2-treated **(right)** PMCA samples before (–) and after (+) being digested with proteinase K. **(B)** Th-T fluorescence of different PMCA cycles of treated (green) and untreated (black) samples. Soluble α-Syn and PMCA steps 4 and 5 are shown. Th-T fluorescence is plotted as normalized means. Error bars are shown as standard errors of mean values.

Protein misfolding cyclic amplification assays, initially developed to study the polymerization and propagation process of the prion protein (Barria et al., 2012; Morales et al., 2012), has been recently adapted for α-Syn amyloid aggregation (Herva et al., 2014). Essentially, cycles of incubation at 37°C are followed by vigorous sonication in order to allow fibril growth and subsequent fibrillar rupture, thus producing α-Syn seeds. These preformed seeds are used to trigger the aggregation of fresh protein in the following cycle, amplifying the fibrillar content. At 90 μM of α-Syn, PMCA produced amyloid structures resistant to protease K (PK) digestion, as observed by SDS-PAGE, with the maximum protection arising after four rounds (Figure 5A, middle). Th-T fluorescence measurements of the same samples indicated that this protection correlates with an increasing presence of amyloid-like assemblies (Figure 5B). In sharp contrast, in the presence of ZPD-2, the amount of PK-resistant protein after four rounds is negligible (Figure 5A, right), Th-T fluorescence signal being also significantly low relative to control samples at this stage (Figure 5B). These results suggested that ZPD-2 was strongly interfering with the PMCA-promoted seeding of α-Syn amyloids. The fact that Th-T decrease becomes significant only at pass 4, likely indicates that the aggregated non PK-resistant species generated at early steps still retain certain Th-T binding ability, since SDS-PAGE analysis indicates that the levels of PK-resistant protein is already decreased in treated samples at passes 1–3 (Supplementary Figure S3).

### ZPD-2 Prevents the Aggregation of Different α-Synuclein Amyloid Conformations

**FIGURE 6:**
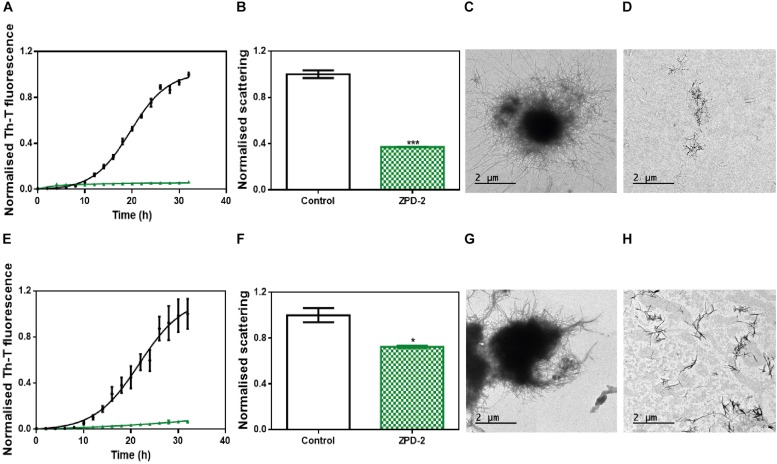
ZPD-2 blocks the aggregation of two different α-synuclein strains. **(A,E)** Aggregation kinetics of α-Syn strains B **(A)** and C **(E)** in absence (black) and presence (green) of ZPD-2. **(B,F)** Light scattering final point measurements at 300 nm of untreated (white) and ZPD-2-treated samples (green) of strains B **(B)** and C **(F)**. **(C,D,G,H)** Representative TEM images of untreated α-Syn aggregates **(C,G)** and treated **(D,H)** samples for strains B and C, respectively. Th-T fluorescence is expressed as normalized means. Final points were obtained at 48 h after the aggregation reaction begins. Error bars are shown as standard errors of mean values, where *p* < 0.05 and *p* < 0.001 were indicated by ^∗^ and ^∗∗∗^, respectively.

The aggregation of α-Syn has been described to lead to the formation of different amyloid conformations, or strains, depending on the environmental conditions (Li et al., 2018); a property that has been linked with its spreading in the brain and the manifestation of different synucleinopathies (Peelaerts et al., 2015). We analyzed the capacity of ZPD-2 to prevent the aggregation of α-Syn into different previously described amyloid conformations (Bousset et al., 2013; Carija et al., 2019). We refer them as strain B (buffer B, 50 mM Tris–HCl pH 7.0) and strain C (buffer C, 50 mM Tris–HCl pH 7.0 supplemented with 150 mM NaCl), to keep the original strain nomenclature. ZPD-2 was active in both cases (Figures 6A,E), inhibiting by up to 90% the formation of the amyloid strains B and C, as monitored by Th-T fluorescence. Light scattering measurements (Figures 6B,F) and TEM imaging (Figures 6C,D,G,H) and soluble protein quantification at the end of the reaction (Supplementary Figure S1B) of the different samples confirmed the inhibitory activity of ZPD-2 against the two strains. Non-fibrillar aggregates might be necessary for fibril formation (obligate), able to convert into fibrils, but not indispensable for fibril formation (on-pathway), or unable of converting directly to fibrils (off-pathway). The difference between the large reduction in Th-T fluorescence promoted by ZPD-2 in strain C aggregation kinetics and the moderate impact the molecule has in light scattering and soluble protein levels might indicate the formation of Th-T negative off-pathway aggregates in these conditions, since they do not evolve into fibrils. However, their size should be rather small, since we did not observe any large amorphous aggregate in ZPD-2-treated samples (Figure 6H).

**FIGURE 7:**
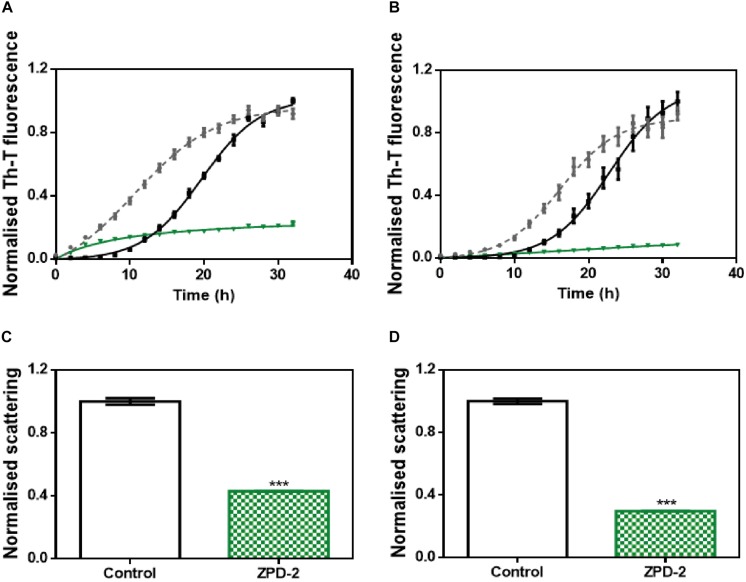
Seeding assays with three different strains. **(A,B)** Aggregation kinetics of α-Syn, buffer B (50 mM Tris–HCl pH 7.0) **(A)**, or buffer C (50 mM Tris–HCl pH 7.0 supplemented with 150 mM NaCl) **(B)**, reported by Th-T fluorescence, in absence of compounds and seeds (black), in presence of 1% (v/v) of preformed seeds at the specific condition (gray dotted line) and in presence of seeds and 100 μM of ZPD-2 (green). Light dispersion of treated (green) and untreated (white) seeded samples at final point of strain B **(C)** and strain C **(D)**. Error bars are shown as standard errors of mean values, ^∗∗∗^*p* < 0.001.

We addressed whether the strong inhibitory capability of ZPD-2 at neutral pH can be overridden by the presence of preformed fibrils able to seed the aggregation reaction. The addition of 1% (v/v) of seeds effectively accelerated the formation of both B and C strains (Figures 7A,B). However, the presence of ZPD-2 abrogates this effect, reducing the final amount of amyloid-like structures in seeded reactions by an 87% for strain B (Figure 7A) and a 90% for strain C (Figure 7B), according to Th-T fluorescence. Again, light dispersion measured at 300 nm revealed a significant decrease of aggregates by 57 and 70% in the case of strains B and C, respectively (Figures 7C,D).

### ZPD-2 Reduces the Formation of α-Synuclein Inclusions in a *C. elegans* Model of PD

**FIGURE 8:**
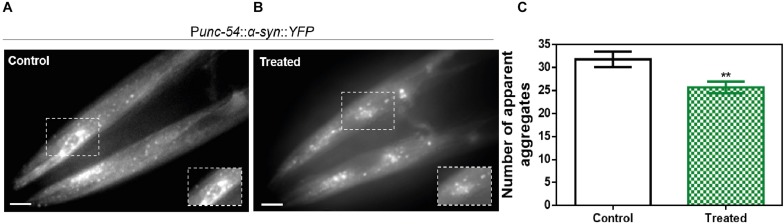
*In vivo* anti-aggregational assays in *Caenorhabditis elegans*. Representative images of apparent α-Syn aggregates in *C. elegans* body wall muscle cells obtained by epifluorescence microscopy of NL5901 worms treated without **(A)** and with ZPD-2 **(B)**. **(C)** Quantification of α-Syn muscle inclusions in the absence (white) and presence of ZPD-2 (green). ^∗∗^*p* < 0.01.

We assessed the toxicity of ZPD-2 for human neuroblastoma cells. No significant toxicity was observed when the molecule was added to the cell culture up to 80 μM (Supplementary Figure S4). We skipped efficacy studies on neuroblastoma cells, because, with more than 20 different compounds analyzed, we could not find a straightforward connection between the potency of the molecules in cell cultures and that in our *C. elegans* models of PD. We first analyzed the effect of ZPD-2 in the *C. elegans* strain NL5901. This strain over-expresses human α-Syn fused to the yellow fluorescent protein (YFP), under the control of the muscular *unc-54* promoter, transgene *phIs2386* (*Punc-54:*α*-SYN:YFP*). The expression of human α-Syn in the muscle of this nematode has been successfully used to identify modifier genes (Hamamichi et al., 2008; van Ham et al., 2008). Animals at the fourth larval stage (L4) were incubated in the presence or absence of 10 μM ZPD-2 and analyzed at 9 days post-hatching (L4 + 7). These aged worms, which mimic aged PD patients, were then analyzed by epifluorescent microscopy and the number of visible α-Syn inclusions was quantified (Figures 8A,B). In these assays, ZPD-2 moderately, but significantly, reduced the number of apparent aggregates (25.7 ± 1.3) when compared to untreated worms (31.8 ± 1.7) (Figure 8C). In addition, worms treated with ZPD-2 showed an increase in their mean lifespan of 14.2%, relative to untreated animals (*p*-value = 0.015, Wilcoxon unpaired test) (Supplementary Figure S5).

### Neuroprotective Role of ZPD-2 in a *C. elegans* Model of PD

**FIGURE 9:**
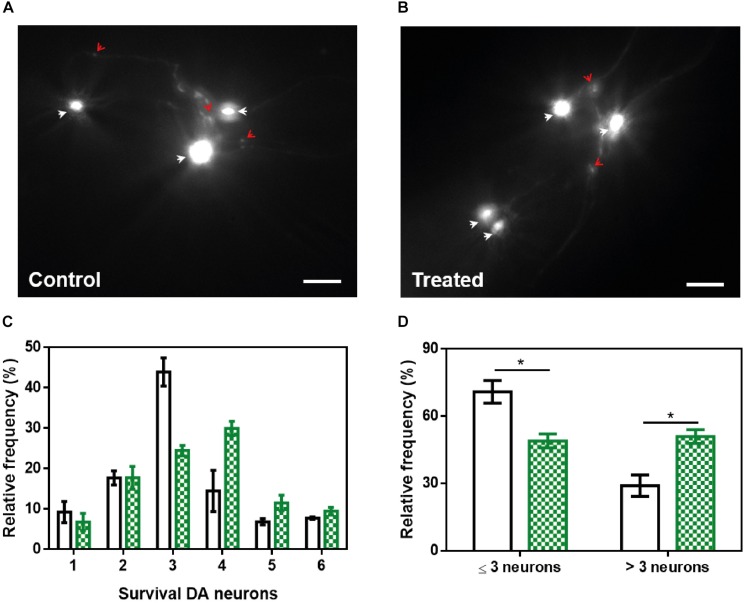
Neuroprotective activity of ZPD-2 in a *Caenorhabditis elegans* model of PD. Representative images of GFP and α-Syn expressing anterior DA neurons in worms treated without **(A)** and with ZPD-2 **(B)** for 7 days after L4. Healthy neurons are labeled with white arrows. **(C)** Distribution of DA surviving neurons in the anterior region of worms. **(D)** Percentage of worms containing three neurons functional or less and more than three functional neurons after 7 days post-hatching. White bars indicate control samples while the green ones correspond to treated samples. ^∗^*p* < 0.05.

The loss of DA neurons is one of the most important characteristics of PD and an important target in the search for a future treatment for this disorder. *C. elegans* presents a total of four pairs of DA neurons, three of them in the anterior part (CEPD, CEPV, and ADE) and one pair in the posterior part (PDE) (Sulston et al., 1975). The existence of six anterior DA neurons has been recently used to analyze PD-related processes in a model (strain UA196) that expresses both human α-Syn and GFP under the control of the dopamine transporter promoter (*Pdat-1:GFP; Pdat-1:*α*-SYN*) (Kim et al., 2018). Human α-Syn expression in these DA neurons induces a progressive degeneration process (Cao et al., 2005). At 9 days post-hatching, the number of remaining functional neurons of untreated (Figure 9A) and ZPD-2-treated (Figure 9B) worms was analyzed. As an average, in control worms 48.1% of DA neurons are non-functional, whereas in treated animals this value decreases to 40.4% (*p*-value = 0.038, Wilcoxon unpaired test). Despite the difference between both means is rather low, the distribution of the data indicated a displacement in the DA neurons survival profile (Figure 9C and Supplementary Figure S6) in the presence of ZPD-2 when compared to the control worms. As a result, there is a significant increase in the number of worms containing more than three functional neurons in the anterior region in the presence of ZPD-2 (51.0 ± 4.8%) when compared to the controls (29.1 ± 3.1%) (Figure 9D).

## Discussion

Protein aggregation is tightly connected with neurodegenerative disorders such as Alzheimer’s and PDs. Immediately after the identification of α-Syn as the main fibrillar component in LBs and LNs (Spillantini et al., 1997, 1998) it became evident that targeting the aggregation of this protein might hold therapeutic potential (Tatenhorst et al., 2016).

Nevertheless, the absence of a defined three-dimensional structure for the functional state of α-Syn due to its intrinsically disordered nature makes the rational design of effective inhibitors that stabilize α-Syn and thus prevent or delay its aggregation, as it has been successfully done for globular proteins like transthyretin (Bulawa et al., 2012; Sant’Anna et al., 2016), difficult. In this scenario, evaluation of large chemical libraries appears as one of the few strategies we have to discover an effective inhibitor of α-Syn deposition and, indeed, this approach has already rendered promising molecules (Levin et al., 2014; Moree et al., 2015; Tatenhorst et al., 2016; Perni et al., 2017; Pujols et al., 2018). In the present work, we describe the discovery of ZPD-2, a small molecule able to prevent up to 90% the *in vitro* aggregation of WT α-Syn and of familial mutants of the protein when used in a 0.7:1 (protein:ZPD-2) ratio, delaying also significantly the completion of the reaction. Its inhibitory capacity was confirmed by orthogonal techniques such as light scattering and TEM.

Further analysis demonstrated that ZPD-2 was able to prevent the aggregation in a concentration-dependent manner, with ∼50% inhibition at a 7:1 protein:compound ratio. This, together with solution NMR measurements indicate that ZPD-2 does not interact significantly with soluble monomeric α-Syn, which suggests that it will not interfere with the functional state of the protein. In addition, the inhibitory potential of ZPD is time-dependent, being more significant at early (0–8 h) stages, in fair contrast with SC-D, a compound we identified in the same screening campaign, whose activity was time-independent, being able to target late species (Pujols et al., 2018). The largest affinity of ZPD-2 for early aggregating species is also inferred from the fact that it mainly impacts the nucleation constant, reducing it by threefold. This might also explain why, at a 0.7:1 ratio, the molecule works well for the A30P (96% inhibition) and H50Q (94% inhibition) familial variants, provided that both mutations facilitate oligomerization, H50Q favoring also fibrillation (Marvian et al., 2019).

ZPD-2 is able to inhibit the aggregation of α-Syn under different solution conditions. This ability opens a possibility for its use in different synucleinopathies, where different α-Syn strains might occur (Bousset et al., 2013; Peelaerts et al., 2015). Importantly, ZPD-2 is one of a few small molecules shown to inhibit efficiently α-Syn seeded aggregation, where the lag phase of the reaction is shortened or abrogated because the soluble protein can be directly incorporated on top of the preformed fibrillar fragments. This seeding-blocking activity explains why ZPD-2 is so effective preventing the formation of PK-resistant/Th-T-positive species in PMCA assays, which promote both templated seeding and aggregates amplification. This effect might respond to the ability of the compound to either destabilize small aggregates or to prevent their elongation, a property that can be very relevant to prevent the cell-to-cell spreading of misfolded α-Syn.

ZPD-2 had not detectable toxic effect for neuronal cells at 10 μM, a concentration at which it reduces the presence of α-Syn inclusions in a *C. elegans* model of PD expressing human α-Syn in body wall muscle cells and extends lifespan. Not surprisingly, this anti-aggregational activity translates in reduced DA neurons degeneration in a *C. elegans* model that over-expresses human α-Syn exclusively in these cells, increasing significantly the proportion of animals that keep > 50% of their anterior part DA neurons intact.

## Conclusion

In conclusion, ZPD-2 properties make this molecule a promising hit for the sake of developing leads able to tackle α-Syn aggregation and seeds propagation in PD and, potentially, other synucleinopathies.

## Data Availability Statement

All datasets generated for this study are included in the article/Supplementary Material.

## Author Contributions

SP-D, JP, XS, JavS, ED, and SV conceived and designed the experiments, and analyzed the results. SP-D, JP, FP, JaiS, and MC-G performed the aggregation assays. AČ expressed and purified the H50Q and A30P variants. SP-D and SN performed the PMCA assays. SN performed the toxicity assays. JG and XS performed the NMR assays. SP-D and ED performed the *C. elegans* tests. SP-D, JP, and SV wrote the manuscript.

## Conflict of Interest

The authors declare that the research was conducted in the absence of any commercial or financial relationships that could be construed as a potential conflict of interest.

## Supplementary Material

FIGURE S1 α-Synuclein soluble fraction at the end of the aggregation. **(A)** Soluble fraction of α-Syn when incubated in absence (black) or presence (green) of ZPD-2 in PBS solution. **(B)** Soluble fraction of strains B and C at final point of the aggregation when incubated in presence (green) or absence (black) of ZPD-2.

FIGURE S2 Lack of interaction between monomeric α-synuclein and ZPD-2 assessed by NMR. Superposition of the 1H-15N HSQC NMR spectra of 15N-labeled α-Syn (70 μM) in absence (black) and presence (green) of 100 μM of ZPD-2.

FIGURE S3 PMCA assay at early stages. Tricine–SDS-PAGE gels of untreated **(middle)** and ZPD-2-treated **(right)** PMCA samples before (−) and after (+) being digested with proteinase K. Soluble α-Syn and PMCA steps 1–3 are shown.

FIGURE S4 Toxicity assays. Analysis of neuronal cells culture survival in presence of different concentration of ZPD-2. Survival is potted as normalized means. Error bars are shown as standard error of means values, where *p* < 0.001 was indicated by ^∗∗∗^.

FIGURE S5 *C. elegans* lifespan analysis. Effect of ZPD-2 treatment (green) on the survival of PD model animals, in comparison with untreated PD worms (black). The data represent the survival ratio (approximately 60–80 animals per group).

FIGURE S6 Distribution of functional neurons in the *C. elegans* dopaminergic model. Normal distribution of the remaining functional dopaminergic (DA) neurons in transgenic animals when treated with ZPD-2 (green) or vehicle (gray). The dashed line delimits animals having four or more functional DA neurons.
