# Supplementary figures and images for: ZPD-2, a Small Compound That Inhibits α-Synuclein Amyloid Aggregation and Its Seeded Polymerization
Source: Front Mol Neurosci. 2019 Dec 17;12:306. doi: 10.3389/fnmol.2019.00306 (PMC6928008; doi:10.3389/fnmol.2019.00306)

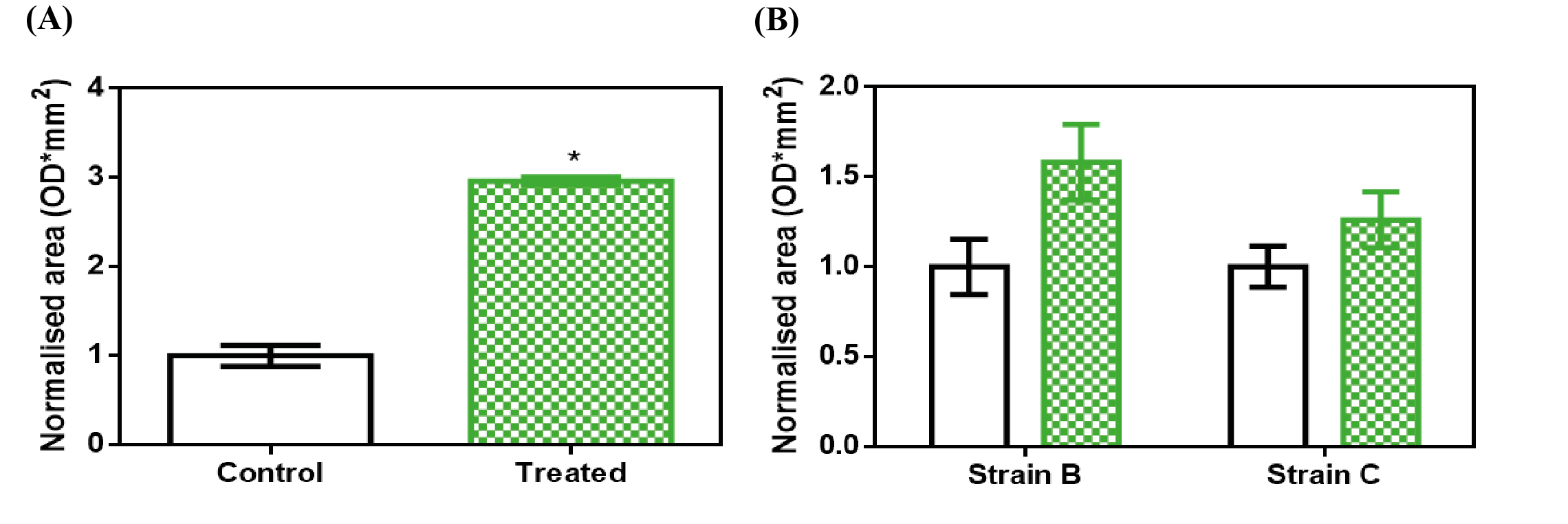

Supplement: FIGURE S1 — α-Synuclein soluble fraction at the end of the aggregation. (A) Soluble fraction of α-Syn when incubated in absence (black) or presence (green) of ZPD-2 in PBS solution. (B) Soluble fraction of strains B and C at final point of the aggregation when incubated in presence (green) or absence (black) of ZPD-2. [file Image_1.JPEG]

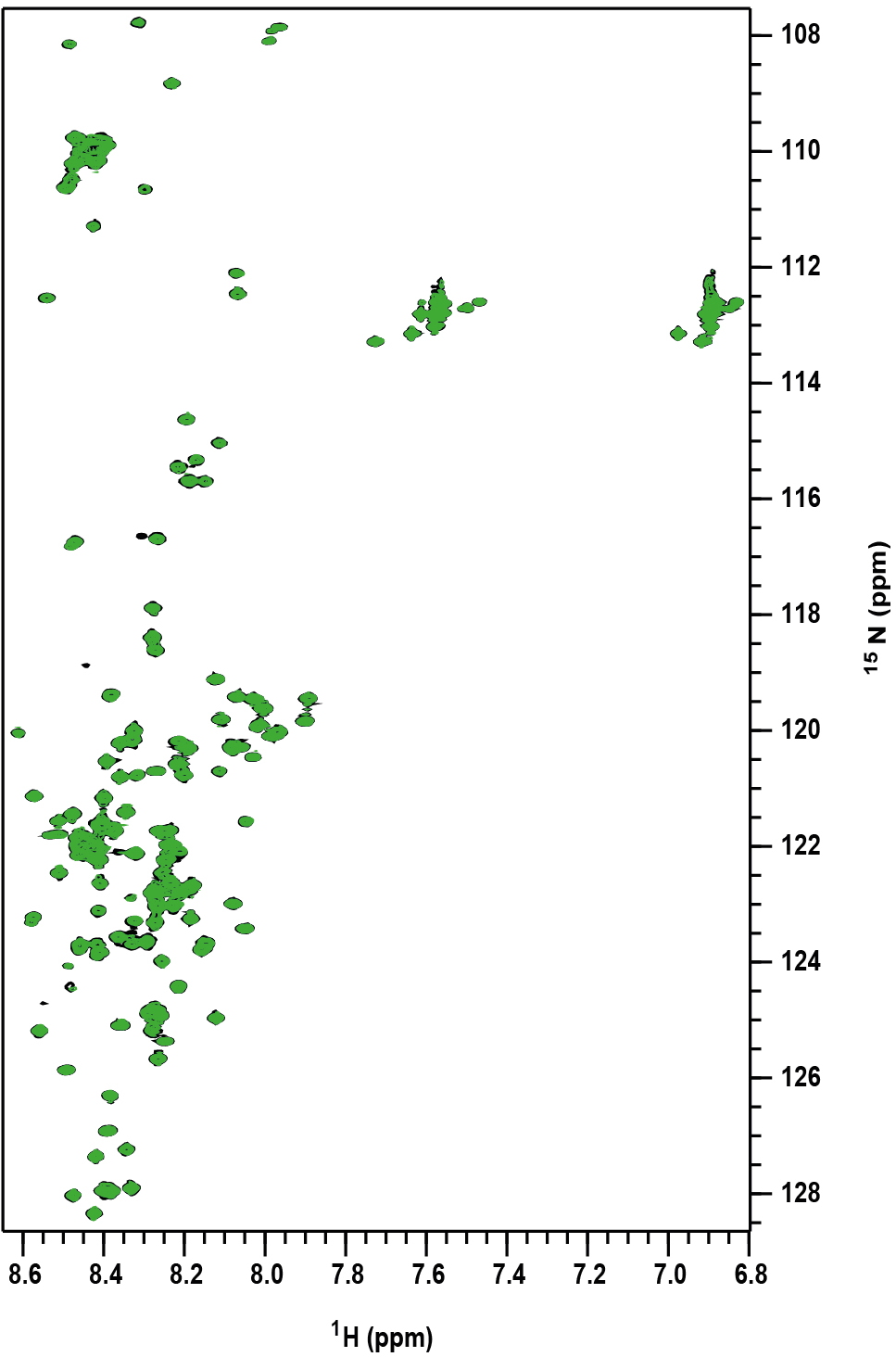

Supplement: FIGURE S2 — Lack of interaction between monomeric α-synuclein and ZPD-2 assessed by NMR. Superposition of the 1H-15N HSQC NMR spectra of 15N-labeled α-Syn (70 μM) in absence (black) and presence (green) of 100 μM of ZPD-2. [file Image_2.JPEG]

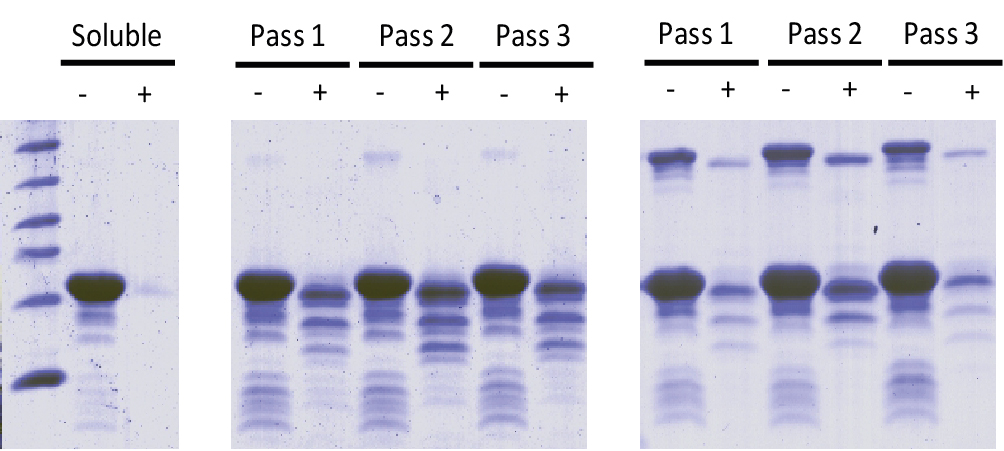

Supplement: FIGURE S3 — PMCA assay at early stages. Tricine–SDS-PAGE gels of untreated (middle) and ZPD-2-treated (right) PMCA samples before (−) and after (+) being digested with proteinase K. Soluble α-Syn and PMCA steps 1–3 are shown. [file Image_3.JPEG]

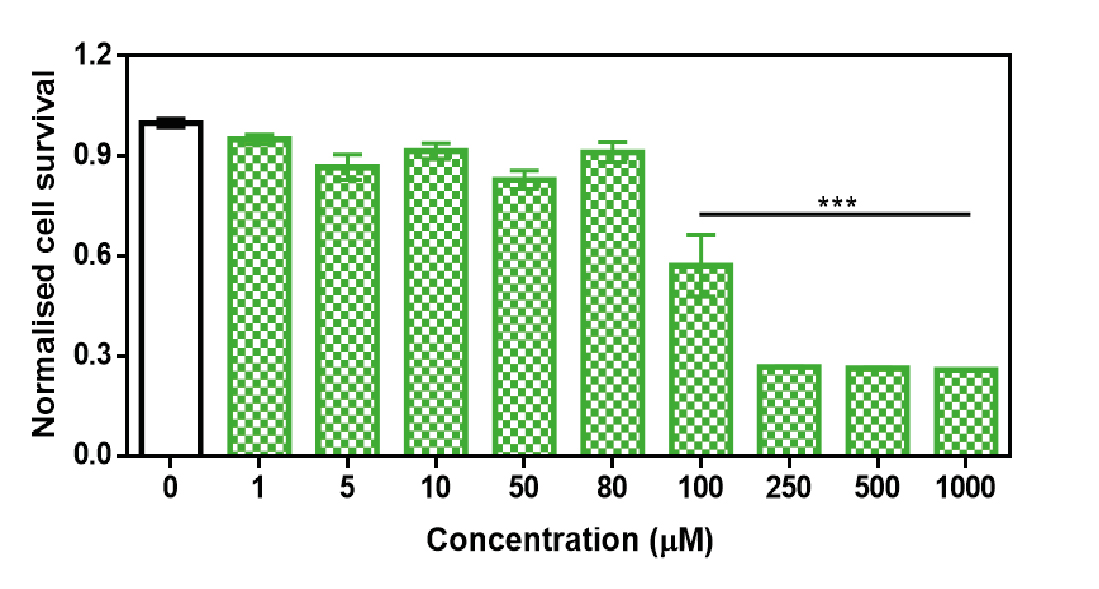

Supplement: FIGURE S4 — Toxicity assays. Analysis of neuronal cells culture survival in presence of different concentration of ZPD-2. Survival is potted as normalized means. Error bars are shown as standard error of means values, where p < 0.001 was indicated by ∗∗∗. [file Image_4.JPEG]

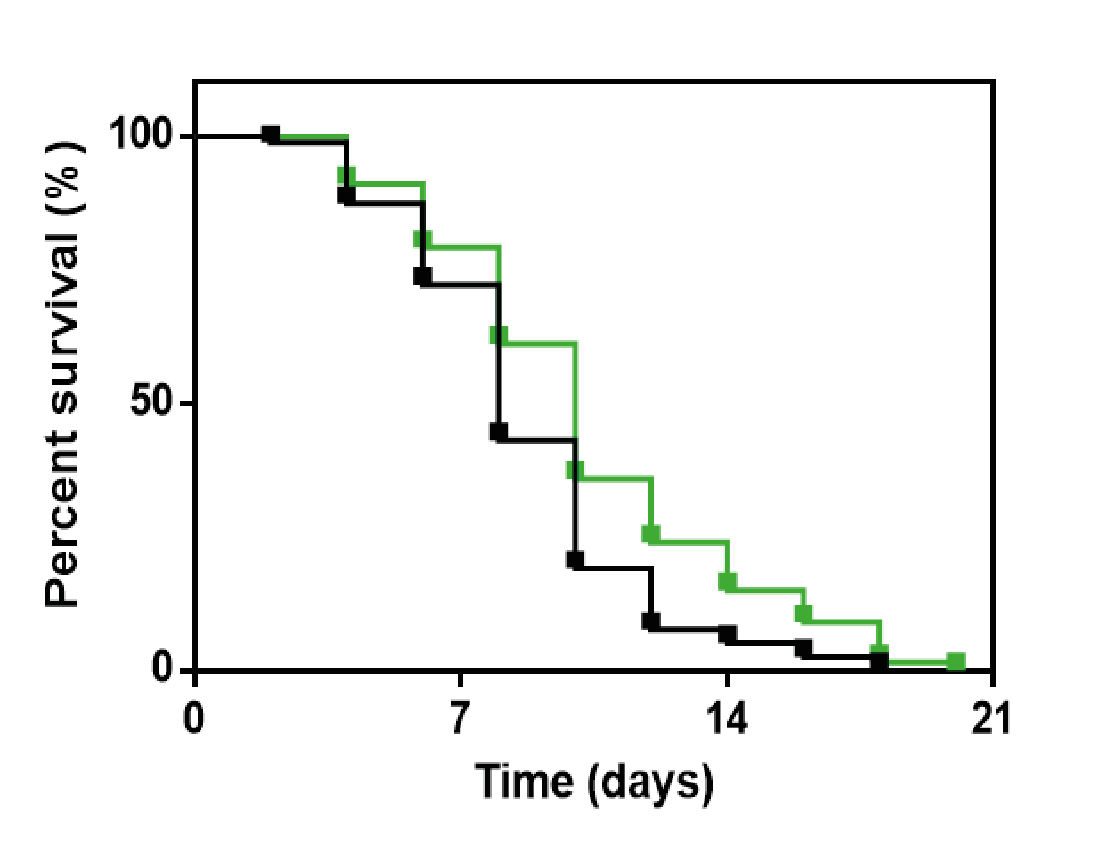

Supplement: FIGURE S5 — C. elegans lifespan analysis. Effect of ZPD-2 treatment (green) on the survival of PD model animals, in comparison with untreated PD worms (black). The data represent the survival ratio (approximately 60–80 animals per group). [file Image_5.JPEG]

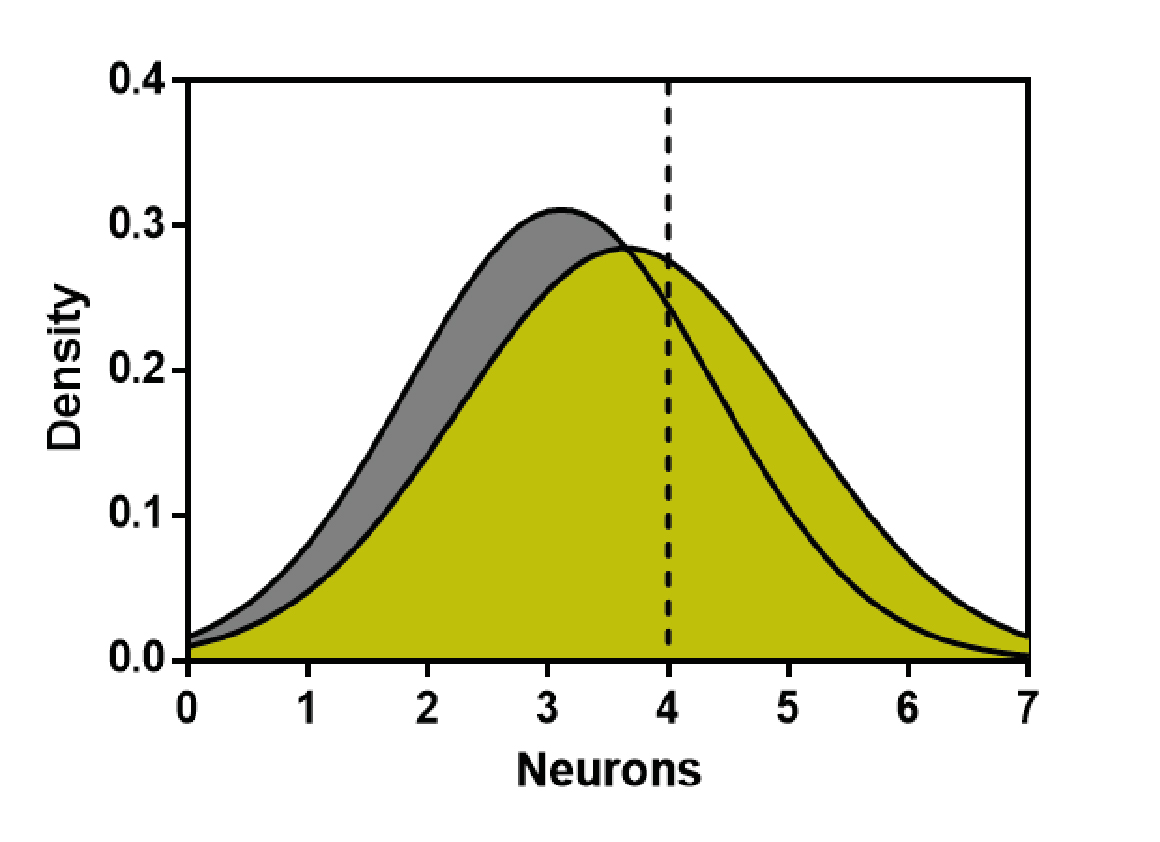

Supplement: FIGURE S6 — Distribution of functional neurons in the C. elegans dopaminergic model. Normal distribution of the remaining functional dopaminergic (DA) neurons in transgenic animals when treated with ZPD-2 (green) or vehicle (gray). The dashed line delimits animals having four or more functional DA neurons. [file Image_6.JPEG]
